# New Record of Spiders (Arachnida: Araneae) from Bashagard District, Southern Iran, with the Report of a Medically Important Species

**DOI:** 10.1155/2022/9509404

**Published:** 2022-05-26

**Authors:** Amin Hosseinpour, Seyed Aghil Jaberhashemi, Mozaffar Vahedi, Aboozar Soltani

**Affiliations:** ^1^Department of Medical Entomology and Vector Control, School of Health, Shiraz University of Medical Sciences, Shiraz, Iran; ^2^Student Research Committee, Department of Medical Entomology and Vector Control, School of Health, Shiraz University of Medical Sciences, Shiraz, Iran; ^3^Research Center for Health Sciences, Institute of Health, Department of Medical Entomology and Vector Control, School of Health, Shiraz University of Medical Sciences, Shiraz, Iran

## Abstract

Spiders are the largest order of arachnids with some medically important species. Considering that no comprehensive research has been conducted on the fauna and distribution of Araneae in Bashagard County (Hormozgan Province) so far, the present investigation has been carried out on these essential issues in this deprived tropical region. Fifteen sampling locations were selected based on the extent of each area and considering climatic characteristics. Samplings were carried out at least four times in each region from February 2017 to September 2018, covering up to a radius of 500 meters from the defined locations. Specimens were collected using the hand collection method, pitfall trapping, and aspirator sampling. All collected samples were preserved in 70% ethanol and were identified using valid taxonomic keys. Of all the 390 collected samples, 134 identifiable specimens were considered for morphological identification. 11 species belonging to 10 genera and seven families were identified. Immature specimens were identified only at the family level. *Wadicosa fidelis* was the most distributed and abundant species in the area, with 13 localities and 84.33% of all identifiable samples. *Plexippus minor* is a new record for the spider fauna of Iran. Moreover, *Loxosceles rufescens*, a medically important species, *Artema doriae*, and *Eusparassus mesopotamicus* were reported for the first time from Hormozgan province. Despite collecting only one specimen, of *L. rufescens*, due to the lack of proper medical facilities and transportation systems in the area, health staff must be alert about this medically important species and warn residents about its potential dangers.

## 1. Introduction

Araneae (spiders) is one of the largest orders of Arachnida, and they are found worldwide on all continents except Antarctica. They generally inject venom into other animals, including humans, to protect themselves or hunt their host [1, 2]. Spiders consist of 49937 species, classified into 130 families and 4239 genera [3]. Of these, four genera *Loxosceles* Heineken and Lowe, 1832 (Sicariidae), *Latrodectus* Walckenaer, 1805 (Theridiidae), *Phoneutria* Perty, 1833 (Ctenidae), and *Atrax* O. Pickard-Cambridge, 1877 (Atracidae), are the most medically important spiders that bite and are dangerous to humans. However, most cases of the spider bite effect are benign, and in some cases, it may lead to serious consequences. In the Asia-Pacific region, several medically important spiders may cause either malignant systemic clinical envenomation syndromes or significant local reactions [4].

**Figure 1:**
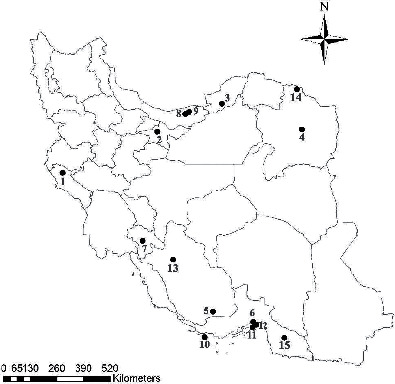
Geographical distribution map of *Loxosceles rufescens* in Iran. 1: Ilam Province (*Loxosceles* sp) (14). 2: Tehran Province (35°43′N, 51°25′E), (9). 3: Golestan Province (36°50′19″N, 54°26′05″E), (15). 4: Khorasan Province (36°16′24 95″N, 59°34′ 75″E), (11). 5: Fars Province, Larestan (27°32′ 44″N, 55°20′ 21″E), (16). 6: Hormozgan Province, Bandar Abbas (27°11′0″N, 56°16′36.0″E),(7). 7: Kohgiluyeh and Boyer-Ahmad Province, road to Choram (30°28′ N, 50°50′ E), (17). 8: Mazandaran Province, Qaem Shahr, (18). 9: Mazandaran Province, Sari, Esfivard-e Shurab, Kord Kheil (36°30′N, 53°00′E), (18). 10: Hormozgan Province, Kish Island, (26°32′N, 54°01′E), (10). 11: Hormozgan Province, Qeshm Island, (26°55′N, 56°08′E), (10). 12: Hormozgan Province, Hormuz Island, (27°04′N, 56°28′E), (10). 13: Fars Province, Shiraz, (29°45′N, 52°45′E), (10). 14: Razavi Khorasan Province, Dargaz, (37°26′2.62″N, 59°06′29.5″E) (19). 15: Hormozgan Province, Bashagard county, Gorichi, (26°49′73″N, 57°63′95″E) present study.

Recluse spider (*Loxosceles* spp.) envenomation is one of the most common health problems in Northern America, Mexico, and Brazil. The genus *Loxosceles* Heineken and Lowe currently consists of 140 species in the world [3]. In Iran, 763 species from 296 genera and 51 families have been reported, and *Loxosceles persica* Ribera and Zamani, 2017, *L. rufescens* (Dufour, 1820), *L. turanensis* Zamani, Mirshamsi, and Marusik, 2021 and *L. coheni* Zamani, Mirshamsi, and Marusik, 2021 have been introduced before from different parts of Iran [5, 6] (Figure 1).

Some cases of loxoscelism caused by *Loxosceles rufescens* have been reported from Turkey, the northwestern neighbor of Iran, and Bandar Abbas, the capital of Hormozgan Province [7]. Moreover, latrodectism caused by *Latrodectus cinctus* bite was reported in southeast Iran [8].

Studies show that the only species of recluse spiders occurring in Iran is the Mediterranean recluse spider, *Loxosceles rufescens*, which has been recorded from Fars, Hormozgan, Razavi Khorasan and Tehran Provinces so far [7]. According to studies conducted in Hormozgan Province, 49 species from 38 genera belonging to 16 families have been identified so far [6].

## 2. Objectives

Despite case reports of spider bites in Hormozgan Province [7] and other neighboring areas [8], so far no comprehensive study on the fauna of spiders has been conducted in Bashagard county. Given the Oriental climate and the occupation of most people in this region (animal farming and agriculture), the possibility of residents' exposure to spiders will increase. Therefore, conducting such studies on medically important spiders in this tropical and lesser-known region seem completely necessary.

## 3. Materials and Methods

**Figure 2:**
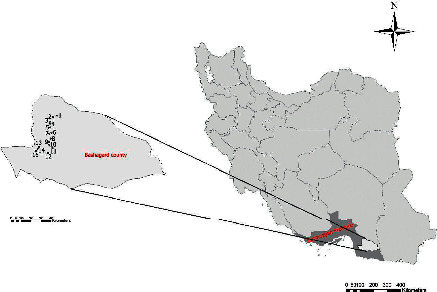
Sampling locations in Bashagard county, Hormozgan province, south of Iran. 1: Irmish, 2: Kalaho, 3: Shahrshib Kala, 4: Irar, 5: Ashkan, 6: Goharan, 7: Pahtek, 8: Posht-e Gar, 9: Babak, 10: Biskav, 11: Sardasht, 12: Dehander-e Shababak, 13: Dargazan, 14: Gorichi, 15: Jakdan.

Bashagard county is located in Hormozgan Province, south of Iran (Figure 2). The average altitude in this county is 2320 above sea level. This study was conducted from February 2017 to September 2018.

Due to different climatic characteristics and a variety of weather conditions in this county, a total number of 15 sampling locations were selected based on the extent of each region (Figure 2). Samplings were carried out at least four times in each area (two times in the day and two times in the night), covering up to a radius of 500 meters from the defined locations.

Specimens were collected mainly from inside and surroundings of different ecosystems and residential areas, using various methods, including hand collection, pitfall trapping, and aspirator sampling. All collected samples were preserved in 70% ethanol for further studies.

The epigyne of the female specimens was separated, cleared, and cleaned with 10% KOH solution. Illustrations were captured using an Olympus SC100 camera attached to an Olympus SZ61 stereomicroscope. All spiders were placed in the Entomological Museum of Shiraz University of Medical Sciences (EMSUMS). Nomenclature and global distribution data are according to the World Spider Catalog (2022), previous Iranian records are according to Zamani et al. (2021), and mentioned as “Iranian provinces” [3, 6].

Mr. Alireza Zamani has performed the final confirmation of the morphological identification of all collected species.

## 4. Results

During the sampling period, 391 samples were collected from all defined locations of the county. Of all the collected samples, 127 identifiable specimens were considered for morphological identification. The rest of the samples were unrecognizable because they were juvenile or damaged; they did not have the required morphological characters. 11 species belonging to 9 genera and seven families were identified using valid taxonomic keys (Table 1). Immature specimens were identified only at the family level (Table 2).

**Figure 3:**
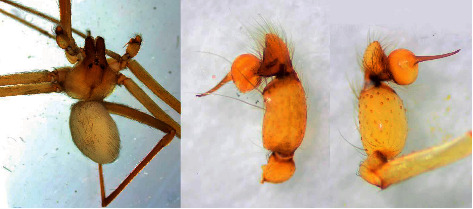
*Loxosceles rufescens* spider, collected in Bashagard, Hormozgan Province, south of Iran. (a) Dorsal view and (b) Palpa. Original photo was taken by A. Hosseinpour.

Out of 15 sampling sites, spider specimens were collected from all locations with different frequencies (Table 1). The most species richness was observed in Location 11 (Sardasht) with seven collected species. Furthermore, three species were also collected from locations 1 and 13 (Irmish, and Dargazan). Two species were collected in locations 2, 4, 9, and 15. Only one species was caught in other sites. *Loxosceles rufescens* (Figure 3), as one of the most medically important species, was collected only from the southwest part of the county in Gorichi (Location 14).

The most frequent species in the region was *Wadicosa fidelis* (O. P.-Cambridge, 1872), with 84.33% of the total collected samples. This species had also the widest distribution among all species in the study area and was collected from 13 sampling locations (out of 15). Additional information on all identified species is provided in detail in the following section.

### 4.1. Family Salticidae Blackwall, 1841

#### 4.1.1. *Plexippoides flavescens* (O. P.-Cambridge, 1872)

Material: *Hormozgan*: 1♂ (EMSUMS), Iran, 26°67ʹ95ʺN, 57°89ʹ91ʺE, July 2017.

Distribution: Greece to Central Asia, Pakistan, and Sudan. Iranian provinces: Alborz, Chaharmahal and Bakhtiari, Fars, Hormozgan, Isfahan, Kerman, Kermanshah, Khuzestan, Kohgiluyeh and Boyer-Ahmad, Lorestan, Mazandaran, Razavi Khorasan, Tehran, and Zanjan.

#### 4.1.2. *Plexippus paykulli* (Audouin, 1826)

Material: *Hormozgan*: 1♂ (EMSUMS), Sardasht, 26°45′61″N, 57°89′78″E, July 2017.

Distribution: Africa. Introduced to both Americas, Europe, Middle East, India, China, Japan, Korea, Philippines, Papua New Guinea, Australia, and Pacific Island. Iranian provinces: Golestan, Hormozgan, Isfahan, Kerman, Khuzestan, Mazandaran, and Tehran.

#### 4.1.3. *Plexippus minor* Wesołowska and van Harten, 2010

Material: *Hormozgan*: 1♂ (EMSUMS), Babak, 26°50′34″N, 57°88′59″E, August 2017.

Distribution: United Arab Emirates.

### 4.2. Family Lycosidae Sundevall, 1833

#### 4.2.1. *Hogna effera* (O. Pickard-Cambridge, 1872)

Material: *Hormozgan*: 1♂ (EMSUMS), Dargazan, 26°47′73″N, 57°77′95″E, July 2017; 1♂ (EMSUMS), Sardasht, 26°45′67″N, 57°89′79″E, June 2017.

Distribution: Greece (Crete), Cyprus, Egypt, Israel, Lebanon, Syria, Lebanon, Syria, Yemen (Socotra), Saudi Arabia, United Arab Emirates, and Iran. Iranian provinces: Alborz, East Azerbaijan, Fars, Hormozgan, Isfahan, Kerman, Kurdistan, Mazandaran, Semnan, and Tehran.

#### 4.2.2. *Wadicosa fidelis* (O. P.-Cambridge, 1872)

Material: *Hormozgan*: 1♀1♂ (EMSUMS), Biskav, 26°48′23″N, 57°87′35″E, August 2017; 3♀ (EMSUMS), Ashkan, 26°65′54″N, 57°88′27″E, September 2017; 3♀ (EMSUMS), Babak, 26°50′30″N, 57°88′49″E, August 2017; 1♂ (EMSUMS), Shahrshib Kala, 26°70′10″N, 57°87′96″E, September 2017; 4♀6♂ (EMSUMS), Irar, 26°67′93″N, 57°89′97″E, July 2017; 3♀2♂ (EMSUMS), Irmish, 26°74′77″N, 57°95′30″E, August 2017; 12♀10♂ (EMSUMS), Kalaho, 26°74′19″N, 57°91′94″E, May 2017; 1♀ (EMSUMS), Kalaho, 26°74′14″N, 57°91′94″E, August 2017; 2♀5♂ (EMSUMS), Goharan, 26°60′47″N, 57°88′99″E, June 2017; 4♀2♂ (EMSUMS), Shahrshib Kala, 26°70′19″N, 57°87′80″E, September 2017; 7♀3♂ (EMSUMS), Dehander-e Shababak, 26°24′69″N, 57°50′63″E, June 2017; 1♂ (EMSUMS), Sardasht, 26°45′67″N, 57°89′79″E, May 2017; 16♀1♂ (EMSUMS), Sardasht, 26°45′67″N, 57°89′79″E, June 2017; 5♀5♂ (EMSUMS), Posht-e Gar, 26°54′00″N, 57°90′09″E, July 2017; 8♀ (EMSUMS), Pahtek, 26°59′26″N, 57°87′86″E, June 2017; 6♀1♂ (EMSUMS), Jakdan, 26°43′92″N, 57°74′72″E, May 2017.

Distribution: Macaronesia, North Africa, Southern Europe, Caucasus, Middle East, Central Asia, China, Japan, Pakistan, India, Bangladesh, Philippines, and Indonesia (Sumatra). Iranian provinces: Golestan, Hormozgan, Ilam, Kermanshah, Khuzestan, Kohgiluyeh and Boyer-Ahmad, Kurdistan, and Razavi Khorasan.

### 4.3. Family Gnaphosidae Pocock, 1898

#### 4.3.1. *Pterotricha strandi* Spassky, 1936

Material: *Hormozgan*: 1♀ (EMSUMS), Sardasht, 26°45′71″N, 57°90′54″E, May 2017; 1♀ (EMSUMS), Jakdan, 26°43′92″N, 57°74′74″E, May 2017.

Distribution: Iran, Afghanistan, Turkmenistan, and India. Iranian provinces: Bushehr, Fars, Hormozgan, Isfahan, Kerman, Kohgiluyeh and Boyed-Ahmad, and Tehran.

### 4.4. Family Oecobiidae Blackwall, 1862

#### 4.4.1. *Oecobius putus* O. P.-Cambridge, 1876

Material: *Hormozgan*: 2♂ (EMSUMS), Dargazan, 26°47′73″N, 57°77′95″E, July 2017.

Distribution: Egypt, Sudan to Iran, Azerbaijan, and India. Introduced to USA and Mexico. Iranian provinces: Fars, Hormozgan, Khuzestan, and Tehran.

### 4.5. Family Sparassidae Bertkau, 1872

#### 4.5.1. *Eusparassus mesopotamicus* Moradmand and Jäger, 2012

Material: *Hormozgan*: 1♂ (EMSUMS), Dargazan, 26°47′73″N, 57°77′95″E, July 2017.

Distribution: Iraq, Iran, and Turkey. Iranian provinces: Khuzestan, Kurdistan, and West Azerbaijan.

#### 4.5.2. *Eusparassus xerxes* (Pocock, 1901)

Material: *Hormozgan*: 1♂ (EMSUMS), Irmish, 26°74′69″N, 57°95′24″E, August 2017; 1♂ (EMSUMS), Sardasht, 26°45′65″N, 57°89′82″E, September 2017.

Distribution: United Arab Emirates, Iran, and Pakistan. Iranian provinces: Bushehr, Hormozgan, Kerman, and Sistan and Baluchistan.

### 4.6. Family Pholcidae C. L. Koch, 1850

#### 4.6.1. *Artema doriae* (Thorell, 1881)

Material: *Hormozgan*: 1♂ (EMSUMS), Sardasht, 26°45′65″N, 57°89′82″E, Septamber 2017.

Distribution: Turkey, Israel, United Arab Emirates, Iran, and Afghanistan. Introduced in Japan. Iranian provinces: Alborz, Fars, Hamedan, Isfahan, Kerman, Kermanshah, Khuzestan, Kohgiluyeh and Boyer-Ahmad, Lorestan, Mazandaran, Razavi Khorasan, Semnan, Tehran, Yazd, and Zanjan.

### 4.7. Family Sicariidae Keyserling, 1880

#### 4.7.1. *Loxosceles rufescens* (Dufour, 1820)

Material: *Hormozgan*: 1♂ (EMSUMS), Gorichi, 26°49′73″N, 57°63′95″E, August 2017.

Distribution: Southern Europe and northern Africa to Iran. Introduced to USA, Mexico, Macaronesia, South Africa, India, China, Japan, Korea, Laos, Thailand, Australia, and Hawaii. Iranian provinces: Alborz, Fars, Hormozgan, Kohgiluyeh and Boyer-Ahmad, Qom, Razavi Khorasan, Mazandaran, and Tehran.

## 5. Discussion

Based on our results, *Plexippus minor* Wesołowska and van Harten, 2010, is a new record for the spider fauna of Iran, and had the northernmost locality in the whole known distribution range. Moreover, in this study, we are reporting *Eusparassus mesopotamicus* Moradmand and Jäger, 2012, and *Artema doriae* (Thorell, 1881) for the first time from Hormozgan Province.

One of the most important species of medical importance, *L. rufescens* (Dufour, 1820) from the family Sicariidae Keyserling, 1880, has been reported from Tehran province for the first time in Iran by Zamani and Rafinejad [9]. In addition, in Hormozgan province, this species has been reported from Kish Island, Bandar Abbas County, Hormuz Island, and Qeshm Island [10].

Several cases of loxoscelism have been reported from different parts of Iran including Hormozgan Province that reflect these arachnids' importance in public health [7, 11, 12].

Recently, latrodectism was reported from the vicinity of Bashagard County (Zahedan, Sistan, and Baluchestan province) [8]. Although we did not catch spiders from this family in this study, the existence of this spider is probable due to its great climatic and geographical similarity with Sistan and Baluchestan province.

In a faunistic study on the spiders of Kohgiluyeh and Boyer-Ahmad Province, 49 species belonging to 38 genera and 15 families were reported [13]. Considering that our study only covered one county, identifying 15 species of spiders is not far from expected. Although biodiversity was not high in our study area, identifying medically important species can be essential and alarming for the area's general public and health policymakers.

## 6. Conclusion

In this study, we increased our knowledge of the geographical distribution of spiders in this lesser-known region. Although most of the specimens caught in this study were spiders with no medical significance, *L. rufescens* is a species with high medical importance. We report its presence from Bashagard county for the first time. Symptoms of loxoscelism ranges from skin necrosis to death. Therefore, it is suggested high-risk people such as farmers and ranchers should be trained on exposure routes and medical intervention after the bite of this spider. As the studied area (Bashagard county) is one of the areas that are difficult to access and does not have access to good connecting roads to transfer the patient to hospitals. In addition, there is not any specialized hospital near the county, it is crucial that health center staff should be trained on the symptoms of the bite and how to treat it. They should be alert about this species and warn high-risk residents about the potential consequences of this spider, especially where the spider was reported from (Location 14: Gorichi).

## Figures and Tables

**Table 1:** Frequency of all collected spider species from each family in Bashagard, south of Iran.

Family	Species	Distribution in 15 locations	Number of samples	Sex
Salticidae Blackwall, 1841	*Plexippoides flavescens* (O. P.-Cambridge, 1872)	1	1	M
	*Plexippus paykulli* (Audouin, 1826)	1	1	M
	*Plexippus minor* (Wesołowska & van Harten, 2010)	1	1	M

Lycosidae Sundevall, 1833	*Hogna effera* (O. Pickard-Cambridge, 1872)	2	2	M
	*Wadicosa fidelis* (O. P.-Cambridge, 1872)	13	113	M/F

Gnaphosidae Pocock, 1898	*Pterotricha strandi* Spassky, 1936	2	2	F

Oecobiidae Blackwall, 1862	*Oecobius putus* (O. P.-Cambridge, 1876)	1	2	M

Sparassidae Bertkau, 1872	*Eusparassus mesopotamicus* (Moradmand & Jäger, 2012)	1	1	M
	*Eusparassus xerxes* (Pocock, 1901)	2	2	M

Pholcidae C. L. Koch, 1850	*Artema doriae* (Thorell, 1881)	1	1	M

Sicariidae Keyserling, 1880	*Loxosceles rufescens* (Dufour, 1820)	1	1	F
SUM				137

**Table 2:** Frequency of juvenile collected spider species from each family in Bashagard, south of Iran.

Family	Number of samples
Salticidae Blackwall, 1841	53
Lycosidae Sundevall, 1833	142
Gnaphosidae Pocock, 1898	61
Oecobiidae Blackwall, 1862	0
Sparassidae Bertkau, 1872	3
Pholcidae C. L. Koch, 1850	5
Sicariidae Keyserling, 1880	0

## Data Availability

The data used to support the findings of this study are available from the corresponding author upon request.
